# Rapid and Efficient Generation of Stable Antibody–Drug Conjugates via an Encoded Cyclopropene and an Inverse‐Electron‐Demand Diels–Alder Reaction

**DOI:** 10.1002/anie.201712370

**Published:** 2018-02-14

**Authors:** Benjamí Oller‐Salvia, Gene Kym, Jason W. Chin

**Affiliations:** ^1^ Medical Research Council Laboratory of Molecular Biology Francis Crick Avenue Cambridge CB2 0QH UK

**Keywords:** antibody–drug conjugates, bioorthogonal reactions, cyclopropene, drug delivery, protein engineering

## Abstract

Homogeneous antibody–drug conjugates (ADCs), generated by site‐specific toxin linkage, show improved therapeutic indices with respect to traditional ADCs. However, current methods to produce site‐specific conjugates suffer from low protein expression, slow reaction kinetics, and low yields, or are limited to particular conjugation sites. Here we describe high yielding expression systems that efficiently incorporate a cyclopropene derivative of lysine (CypK) into antibodies through genetic‐code expansion. We express trastuzumab bearing CypK and conjugate tetrazine derivatives to the antibody. We show that the dihydropyridazine linkage resulting from the conjugation reaction is stable in serum, and generate an ADC bearing monomethyl auristatin E that selectively kills cells expressing a high level of HER2. Our results demonstrate that CypK is a minimal bioorthogonal handle for the rapid production of stable therapeutic protein conjugates.

Enhancing the selective delivery of drugs to the desired tissues is a prime goal in the development of therapeutics. Antibody–drug conjugates (ADCs) seek to address this need by directing potent cytotoxins to specific cell types, thus minimizing the off‐target effects of traditional chemotherapy. Site‐specific conjugation of drugs to antibodies can improve the therapeutic index with respect to first‐generation heterogeneous ADCs.^1^ Therefore, several strategies have been reported for the selective chemical and enzymatic attachment of drugs to antibodies.^1^, ^2^ However, the positions on the antibody that can be targeted, the stability of the linkage, and/or the extent of drug conjugation are limited.

Genetic‐code expansion enables the site‐specific incorporation of non‐canonical amino acids (ncAA) into proteins in response to a target codon, commonly the amber stop codon, introduced into a gene of interest.^3^ Orthogonal aminoacyl‐tRNA synthetase/tRNA pairs that enable the incorporation of diverse ncAAs have been discovered,^4^ and the site‐specific incorporation of ncAAs bearing bioorthogonal reactive groups provides an attractive, and potentially general, strategy for generating ADCs.^5^ However, the ncAAs that have been incorporated into antibodies so far, which contain ketones or azides, are not optimal for bioconjugation. Ketones^6^ can react with endogenous amines, require low pH (4.5) and long reaction times (60 h); azides^7^ are prone to reduction^8^ and the copper species used to catalyze cycloadditions with alkynes may induce oxidative damage.^9^ Although alternative highly reactive ncAAs based on large, hydrophobic, carbocyclic handles have recently been encoded in an antibody,^10^ the expression system used suffers from very low yields (0.5 mg L^−1^) and these ncAAs may modify the physicochemical properties of the protein.

Here, we describe antibody‐expression systems that utilize the pyrrolysyl‐tRNA synthetase (encoded by *PylS*)/tRNA_CUA_ (encoded by *PylT*) pair to direct the incorporation of a cyclopropene derivative of lysine (CypK, Figure 1 b)^11^ in response to amber codons introduced into antibody genes. We apply these systems to the production of the FDA‐approved trastuzumab antibody, which targets the breast‐cancer marker HER2. We express trastuzumab with CypK at similar levels to the wild‐type antibody. CypK contains a 1,3‐disubstituted cyclopropene, which provides a minimal bioorthogonal handle that undergoes a rapid and selective inverse‐electron‐demand Diels–Alder cycloaddition with tetrazine derivatives to yield dihydropyridazines.^12^ We show that trastuzumab(CypK)_2_ can be covalently linked to monomethyl auristatin E (MMAE), the microtubule polymerization inhibitor used in Adcetris, an FDA‐approved ADC. We show that the resulting dihydropyridazine linkage is stable in serum and we demonstrate the selectivity and potency of trastuzumab(MMAE)_2_.

**Figure 1:**
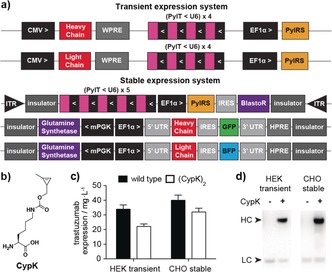
Antibody‐expression systems developed in this study. a) Relevant region of the two plasmids transfected into HEK cells for transient expression and the three components for the stable expression in CHO cells b) *N* ^*ϵ*^‐[((2‐methylcycloprop‐2‐en‐1‐yl)methoxy)carbonyl]‐l‐lysine (CypK) c) Expression yields in the two expression systems. Error bars represent the standard deviation from three biological replicates. d) Western blot shows that the expression of the antibody heavy chain is dependent on the addition of CypK.

To rapidly characterize antibody variants and test the conjugation conditions, we developed a transient expression system, consisting of two plasmids, one encoding trastuzumab heavy chain (HC) and the other encoding the light chain (LC) (Figure 1 a and Figure S1 in the Supporting Information). Previous approaches have relied on a separate plasmid for co‐expression of the expanded‐genetic‐code machinery, *PylS*/*PylT*.^13^ Here we achieve higher antibody yields by transferring *PylT* and *PylS* onto the same constructs as the HC and LC genes and reducing the number of plasmids (Figure S2).

The first position of the CH1 domain on the heavy chain was chosen to encode the ncAA (HC‐118TAG). HC‐118 is permissive for mutation and conjugation^14^ and because it is conserved in all IgG1s the expression system is applicable to most therapeutic monoclonal antibodies. Transfection of the transient plasmid system in Expi293 cells yielded 22±2 mg L^−1^ of trastuzumab(CypK)_2_, which approaches the wild‐type antibody level (33±3 mg L^−1^) (Figure 1 c). We also verified that the expression of HC‐118TAG trastuzumab is ncAA‐dependent (Figure 1 d).

Although the focus of this study was on the incorporation of two drugs per antibody, we also showed the flexibility of the expression system by adapting it to the production of trastuzumab(CypK)_4_. Incorporation of the amber codon at position 107 in the light chain (LC‐107TAG) in addition to HC‐118TAG resulted in trastuzumab with four CypK conjugation sites, one on each HC and LC (Figure S3). This antibody variant is expressed at 10–20 mg L^−1^.

In order to create a robust platform for the expression of antibodies containing ncAAs, we generated a CHO‐S stable cell line in two steps (Figure S4). First, we inserted the genetic‐code‐expansion machinery into the CHO genome by using a piggyback transposase.^15^ Second, we integrated the heavy and light chain genes and selected for high expression of the antibody. After optimizing the CypK concentration in this system (Figure S5), we achieved an antibody expression yield of 31±2 mg L^−1^, 75–80 % with respect to the wild‐type line (Figure 1 c). Prior work suggests that absolute yields can be substantially increased in optimized industrial formats.^16^

No aggregation products were detected by size‐exclusion chromatography after the antibody was concentrated, with protein A resin, from the supernatant. This observation is in line with the bioorthogonality and low hydrophobicity of CypK. Subsequent purification with hydrophobic‐interaction chromatography (HIC) proved sufficient to isolate the pure full‐length antibody. We confirmed incorporation of CypK at position 118 by mass spectrometry (Figure 2 d, and Figures S6 and S7).

**Figure 2:**
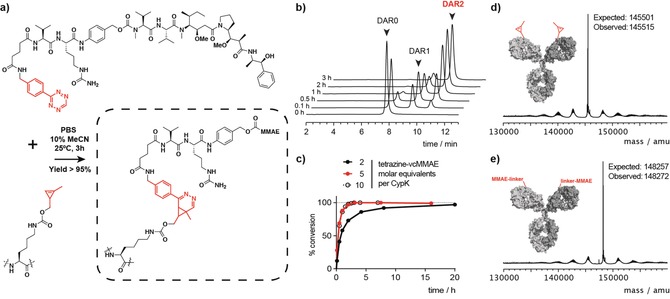
Tetrazine‐vcMMAE conjugation to trastuzumab(CypK)_2_. a) Scheme of the inverse‐electron‐demand Diels–Alder reaction of tetrazine‐vcMMAE with a CypK residue. b) HIC chromatograms showing the evolution of each DAR species with time during the conjugation using five molar equivalents of tetrazine‐vcMMAE per CypK. c) Conversion with respect to maximum DAR (1.9) as measured by HIC‐HPLC. d,e) Deconvoluted mass spectra of deglycosylated trastuzumab(CypK)_2_ and trastuzumab(MMAE)_2_, respectively.

To generate the ADC trastuzumab(MMAE)_2_, we conjugated trastuzumab(CypK)_2_ with a tetrazine‐modified MMAE (tetrazine‐vcMMAE) (Figure 2 a). We included a valine–citrulline protease‐labile component in the linker between the benzyl tetrazine handle and MMAE; upon internalization of the ADC, the dipeptidyl linker is cleaved by cathepsin B in the lysosome to release the toxin.^17^

The cycloaddition reaction between trastuzumab(CypK)_2_ and tetrazine‐vcMMAE was completed within 3 h at 25 °C in phosphate‐buffered saline (PBS; pH 7.4) when five or more equivalents of the toxin per CypK were added (Figure 2 b and c). Due to the high hydrophobicity of the toxin, addition of 10 % acetonitrile is required. This result confirms that CypK allows a substantially faster conjugation than most reported bioorthogonal handles for ADCs. Alternatively, conjugation without acetonitrile and with just two equivalents of peptide per CypK resulted in a completely modified product in 20 h; the low stoichiometry could lead to substantial cost savings during ADC manufacturing in comparison with most existing ncAA‐based technologies. In all cases, a drug‐to‐antibody ratio (DAR) of >1.9 was achieved as measured by LC‐MS and HPLC‐HIC. The identity of the conjugate was verified by LC‐MS (Figure 2 e and Figure S8). Trastuzumab(CypK)_2_ stored for more than four months in PBS at 4 °C maintains its reactivity.

We also conjugated tetrazine‐TAMRA under the same conditions as tetrazine‐vcMMAE, reaching a DAR >1.9 (as measured by LC‐MS) within only 2 h (Figures S9 and S10). Additionally, we showed that the affinity of the antibodies for their antigen is preserved after the conjugation (Figure S11).

The stability of the linker in ADCs is critical because the premature release of the drug results in higher toxicity and lower efficacy.^1^,7c We assessed the integrity of the dihydropyridazine linkage in trastuzumab(TAMRA)_2_ in serum and plasma; since the stability of this linkage can be easily followed by fluorescence and an enzyme‐linked immunosorbent assay (ELISA), and the measurements are not complicated by the protease‐labile component of trastuzumab(MMAE)_2_. The payload remained attached to the immunoconjugate for greater than five days at 37 °C in human serum and plasma (Figure 3 b and Figure S12).

**Figure 3:**
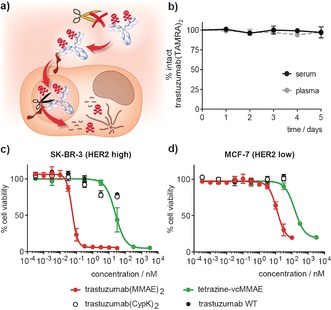
Stability and cytotoxicity of trastuzumab conjugates. a) Schematic of desired properties for internalizing ADCs. b) Stability in human plasma and serum as measured by ELISA. Error bars represent the standard deviation of biological triplicates. c,d) Cell‐viability assay. Data show the mean of three independent experiments and error bars represent the standard deviations.

Finally, to demonstrate the selectivity of trastuzumab(MMAE)_2_, we compared the potency of the ADC in breast‐cancer cell lines expressing high and low levels of HER2. As expected, trastuzuamb(MMAE)_2_ had dramatically increased cytotoxicity with respect to tetrazine‐vcMMAE and the unconjugated antibody in SK‐BR‐3 cells(HER2 high; Figure 3 c and Table S1). The half‐maximal effective concentration (EC_50_) for the MMAE ADC is 55±10 pm, which is consistent with the activity of immunoconjugates obtained using alternative conjugation methods.^18^ By contrast, the EC_50_ of trastuzumab(MMAE)_2_ in MCF‐7 cells, which express 15‐fold fewer HER2 receptors than SK‐BR‐3 cells,^19^ is at least 200 times higher (Figure 3 d and Table S1). These results demonstrate the high selectivity of the ADC for the target cells. We then showed that tetrazine‐vcMMAE and the same toxin without the linker have similar toxicity in the HER2‐high SK‐BR‐3 and the HER2‐low MCF‐7 cell lines (Figure 3 c,d and Figure S13), demonstrating that the antibody is responsible for the aforementioned selectivity. When trastuzuamb(MMAE)_2_ was incubated in human serum for five days prior to its use in the viability assay it retained full toxicity and selectivity, consistent with the conjugate being stable in serum (Figure S14).

In conclusion, we have engineered two mammalian‐expression systems to efficiently and site‐specifically incorporate CypK in trastuzumab. The modified IgG1 can be easily purified and rapidly conjugated with tetrazine‐containing molecules, yielding dihydropyridazine‐linked products that are stable in serum. Finally, we have demonstrated that linking trastuzumab to MMAE by using genetically encoded CypK and bioorthgonal ligation generates a potent and selective ADC. The minimal bioorthogonal handle in CypK may enable facile and rapid conjugation of diverse therapeutic antibodies with sterically hindered third‐generation ADC payloads. The genetic encoding of CypK coupled to its rapid and quantitative conjugation to generate a stable linkage may also prove useful for other protein conjugates intended for therapy and diagnosis.

## Conflict of interest

The authors declare no conflict of interest.

## Supporting information

As a service to our authors and readers, this journal provides supporting information supplied by the authors. Such materials are peer reviewed and may be re‐organized for online delivery, but are not copy‐edited or typeset. Technical support issues arising from supporting information (other than missing files) should be addressed to the authors.

Supplementary
