# Supplementary material for: Rapid and Efficient Generation of Stable Antibody–Drug Conjugates via an Encoded Cyclopropene and an Inverse‐Electron‐Demand Diels–Alder Reaction
Source: Angew Chem Int Ed Engl. 2018 Feb 14;57(11):2831–4. doi: 10.1002/anie.201712370 (PMC5861662; doi:10.1002/anie.201712370)
Supplement: Supplementary file 1 — Supplementary [file ANIE-57-2831-s001.pdf]

Angewandte Chemie
Eine Zeitschrift der Gesellschaft Deutscher Chemiker

Supporting Information

Rapid and Efficient Generation of Stable Antibody–Drug Conjugates via an Encoded Cyclopropene and an Inverse-Electron-Demand Diels–Alder Reaction

*Benjamí Oller-Salvia[+], Gene Kym[+], and Jason W. Chin**

anie_201712370_sm_miscellaneous_information.pdf

Author Contributions

B.O. Conceptualization: Equal; Data curation: Lead; Formal analysis: Lead; Investigation: Equal; Methodology: Equal; Project administration: Supporting; Writing—original draft: Lead; Writing—review & editing: Equal
G.K. Conceptualization: Equal; Data curation: Supporting; Formal analysis: Supporting; Investigation: Equal; Methodology: Equal; Project administration: Supporting; Writing—original draft: Supporting; Writing—review & editing: Supporting
J.C. Conceptualization: Lead; Funding acquisition: Lead; Project administration: Lead; Supervision: Lead; Writing—review & editing: Equal.

Table of contents

Supplementary Materials and Methods....................2

Cell culture conditions for sorting and expression....................2

Plasmid construction....................3

Transient expression....................3

Stable expression cell lines....................4

Expression conditions....................6

Purification....................6

Antibody conjugation and analysis....................7

HER2 binding assay....................9

Stability of the linkage in plasma and serum....................9

Cytotoxicity assay....................10

Supplementary figures and tables....................11

Transient expression system....................11

Stable expression cell lines....................14

Antibody conjugation and analysis....................20

Stability of the linkage in plasma and serum....................24

Cytotoxicity of trastuzumab-MMAE....................25

Trastuzumab sequence....................28

Supplementary References....................29

Supplementary Materials and Methods

Cell culture conditions for sorting and expression. Mammalian CHO and HEK cultures were maintained at 37°C with 8% CO_2 in humidified incubators. Suspension cultures were maintained in the same conditions, but with a shaker at 125 rpm. Expi293F suspension HEK cells were maintained in Expi293 media (Gibco) supplemented with 100 units/mL penicillin, 100 μg/mL streptomycin, and 250 ng/mL Amphotericin B. FreeStyle CHO-S suspension CHO cells were maintained in FreeStyle CHO media (Gibco) supplemented with 8 mM glutamine. CHO-S cells without any integrated plasmids were supplemented with 100 units/mL penicillin and 100 ug/mL streptomycin. CHO-S cells with integrated PylS/PylT genes with blasticidin resistance were supplemented with 1 μg/mL blasticidin. CHO-S cells with PylS/PylT and antibody expression plasmids under glutamine synthetase selection were maintained in media with 1 μg/mL blasticidin but without added glutamine. After FACS, CHO-S cells were grown from single cells in adherent plates without shaking. Blasticidin-resistant cells were grown in Ham's F12 media (Sigma) with 10% FBS, 8 mM glutamine, and 1 μg/mL blasticidin. Blasticidin-resistant and glutamine synthetase selected cells were grown in Ham's F12 media with 10% dialyzed FBS, no added glutamine, and 1 μg/mL blasticidin. Adherent cultures were scaled up from 96 well plate single cells to T-175 flasks, trypsin digested, and transferred to suspension media with the relevant additives as listed before.

Plasmid construction. We cloned transient expression vector pKYM1 (Figure S1a) by replacing the Neomycin resistance cassette in pcDNA3.4 with *Methanosarcina mazei* PylS and 4 copies of $PylT_{U25C}$. We PCR amplified PylS/PylT from our previously reported PiggyBac plasmids.[1] The four copies of PylT are arrayed in tandem, and are placed adjacent to the PylS expression cassette. Because the U6::PylT repeats are difficult to amplify by PCR, we used AccuPrime Pfx polymerase (ThermoFisher) and designed primers to be 300bp away from the start of the PylT cassette.[2] All other PCRs were done with Q5 or Fusion polymerases (NEB). pcDNA3.4, with a multiple cloning site from pcDNA3.1 inserted into the main open reading frame, was linearized by PCR. The two PCR fragments were joined via Gibson assembly into NEB Stable cells and grown at 30°C to reduce recombination of the tRNA repeats. Heavy and light chains were cloned into their own plasmids using Gibson. Trastuzumab genes were ordered as gBlocks from IDT. UAG amber codons were introduced into Gibson homology regions for a multi-piece Gibson assembly.

A PiggyBac plasmid SE315 with 5 copies of $PylT_{U25C}$, an EF1α controlled PylS, and an IRES followed by a blasticidin resistance gene was previously cloned. This plasmid was used to generate amber suppressing CHO-S lines. Trastuzumab HC::IRES::GFP (GFPspark) and LC::IRES::BFP (mTagBFP2) constructs were assembled via PCR and Gibson into separate DNA2.0 stable CHO plasmid pD2531. The HC plasmid was cloned with both wild type and HC-118TAG variants. These plasmids were used to generate stable antibody producing lines. For all plasmids, ncAA and UGA stop codons, often in tandem, were exclusively used at the end of genes. UAG amber codons were only used at sites chosen for incorporation of CypK or BocK.

Transient expression. For transfection, Expi293F cells were grown in Expi293 media without added antibiotics to a density of 2.5×10^6 cells/mL. HC and LC pKYM1 plasmids were mixed 1:1 and transfected using Expifectamine (Gibco) in the presence of CypK. The following day, boosters were added per the manufacturer's instructions. Antibodies were harvested 7 days later from the supernatant.

Stable expression cell lines. Stable cell lines to produce trastuzumab with CypK were generated in two steps: insertion of the genetic code expansion machinery and integration of the heavy and light chains.

In the first step, we generated the amber suppressing lines (CHO-S HC118TAG) by using PiggyBac to insert a cassette with five copies of PylT and PylS under the U6 and EF1α promoters, respectively, into the genome (Figure S4a). SE315, which has 5' and 3' ITRs for integration, was co-transfected with PiggyBac transposase at a 10:1 ratio using PEI into CHO-S cells in suspension culture. A blasticidin resistance marker was included in the vector in order to select for the cells that had efficiently integrated the PylS/PylT cassette (Figure S4b). Two days after transfection, 10 μg/mL of blasticidin was added and the culture was maintained for 10 more days. The batch was grown without blasticidin for one week to recover. Following blasticidin selection, we sought to isolate a clonal line with high amber suppression efficiency. To this end, we transiently transfected a plasmid encoding super-folder green fluorescent protein (sfGFP) with an amber codon at position 150 (CMV-sfGFP150TAG), which only fluoresces when the amber codon is read through. We grew the resulting cells in the presence of 2 mM N^{ε}-(*tert*-butyloxycarbonyl)-L-lysine (BocK) for 4 days with 1:100 anticlumping agent. The batch was filtered to remove clumps and cells with high amber suppression, as indicated by GFP fluorescence, were isolated into 96 well plates using fluorescence-activated cell sorting (FACS).

The efficiency of the selected amber suppressing lines that grew well was verified through rephenotyping using a transient GFP::TAG::mCherry plasmid in adherent cells grown in 2 mM BocK for 4 days. This system allows visualizing relative transfection efficiency through GFP fluorescence and amber suppression efficiency in the mCherry channel (Figure S4c). The best amber suppressing CHO lines were transiently transfected with either the read-through control pCMV-mCherry-GFP or the amber mutant containing a pCMV-mCherry-TAG-GFP reporter. Cells were then grown in no ncAA and analysed after four days on a BD LSR II Flow Cytometer (Figure S4d). A line with a high GFP/mCherry ratio was chosen and scaled up to suspension culture.

In stage two of the cell-line production, we introduced the antibody HC and LC genes in five steps (Figure S4e): insertion, amplification/selection, sorting, manual picking, and small-scale expression tests. First, we transfected trastuzumab HC and LC, each embedded on a separate plasmid (Figure S4f), into the amber suppressing line selected above. We used DNA2.0 plasmids with optimized 5' and 3' untranslated regions (UTRs) around the HC and LC for enhanced expression. pEF1a-Heavychain-ires-GFP and pEF1a-Lightchain-ires-BFP, both on plasmid pD2531 were transfected into amber-suppressing CHO lines using TransIT-PRO transfection reagent. Second, we used the glutamine synthetase marker embedded in the plasmids to amplify the gene through selection with increasing levels of an inhibitor (methionine sulfoximin, MSX) of this essential enzyme. The cells were grown in FreeStyle CHO with no glutamine in the presence of 25 μM MSX for two weeks. They were allowed to recover for one week in media with no glutamine and no MSX. Third, in order to select for cells expressing the highest levels of HC and LC, we used the two distinct fluorescent protein reporters (GFP and BFP) downstream of each antibody chain to isolate the top 2.5% double-positive lines using FACS (Figure S4g). These reporters were preceded by internal ribosome entry sites for co-translational expression with the antibody chains.

Two additional rounds of MSX + FACS selection were performed. Populations from the first round were selected for two more weeks in suspension media with no glutamine and 100 uM MSX, recovered for a week, and the top 1% of double positives were sorted into 96 well plates. Cells were sorted at five cells per well into Ham's F12 with dialyzed FBS (Gibco) without glutamine. Wells with fast-growing colonies that appeared to grow from a single clone (based on number of colonies in the well) were examined by eye for GFP expression under a fluorescent microscope. Ten clones from the wild type antibody and ten from the HC118TAG antibody were scaled up and tested for expression in 2 mM CypK (synthesized as previously described).[3] The best expressing lines were pooled, and underwent another round of MSX amplification at 1 mM for two weeks in glutamine free suspension media. The batch was sorted for top 1% of double positive cells again as stated before. Ten clones from each batch were tested for expression in 2 mM CypK.

Finally, the highest expressing lines that had favorable growth properties such as growth rates and lack of clumping were tested for expression in suspension media with

no glutamine, 1:100 anti-clumping agent (Gibco), and 1 mM BocK (E1610.0025, Bachem). Cultures were started at 10^6 cells/mL and run for 7 days.

Expression conditions. 100 mL of CHO-S HC118TAG suspension cell cultures was treated with 5 mM CypK (from a 100 mM CypK stock in 0.1 M NaOH), 100 units/mL penicillin, 100 ug/mL streptomycin, 250 ng/mL Amphotericin B (Antibiotic-Antimycotic, Gibco), and anti-clumping solution (1:200). The pH of the solution was adjusted to pH 7.4 with HCl 4 M and filtered prior to cell resuspension.

Purification. Cells were centrifuged for 5 min at 250 rcf. The supernatant (SN) was filtered and mixed with 5x bead wash buffer (0.5 M sodium phosphate, 150 mM NaCl). Trastuzumab$(CypK)_2$ was captured with protein A resin (2 mL resin/100 mL SN, Sino Biological), previously equilibrated with bead wash buffer (0.1 M sodium phosphate, 150 mM NaCl), for 3h at room temperature on a tube roller. The beads were washed with 100 mL of bead wash buffer and eluted with 11 mL of 0.1 M sodium citrate pH 3 on 3 mL of 1 M phosphate buffer 150 mM NaCl. Trastuzumab$(CypK)_2$ was concentrated and buffer-exchanged three times with PBS (Amicon Ultra-15, 50K MWCO, Merck Millipore). Samples were purified by FPLC using a butyl HIC column (HiTrap™ 1 mL Butyl HP, ThermoFisher) with a flow of 0.5 mL/min and a 0-100% in 20 min gradient of B (50 mM sodium phosphate pH 7.0, 20% isopropanol) in A (1.5 M $(NH_4)_2SO_4$, 50 mM sodium phosphate pH 7.0, 5% isopropanol). All chromatograms were acquired at 280 nm. Samples below 1 mg were purified by HPLC-HIC using the conditions described in the analytical section. Elution buffer was exchanged for PBS. The antibody was stored at 4ºC.

Quantification was carried out by western blotting in cell supernatants and by coomassie blue staining in samples purified by protein A. In both cases standards were run on the same gels and used in order to generate a calibration curve. Concentration in HIC-purified samples was quantified by UV spectrometry (Nanodrop) applying Lambert-Beer's law (ε = 225000 $M^{-1}\cdot cm^{-1}$, Mw = 148000).

Antibody conjugation and analysis. By default, 10 molar equivalents (eq) of tetrazine-vcMMAE (synthesized by ChemPartner) or tetrazineTAMRA (Jena Bioscience) (1.5 μL, 3.4 mM in DMSO) was diluted with 7.5 μL of acetonitrile and 28.5 μL of PBS. Trastuzumab(CypK)$_2$ (37.5 μL, 2 mg/mL in PBS, 1 eq) was added to the solution and was allowed to react for 3 h at 25°C. When 20 eq of tetrazine-vcMMAE were used (1.5 μL, 6.8 mM in DMSO), this compound was mixed with 15 μL of acetonitrile and 21 μL of PBS, and incubated with trastuzumab(CypK)$_2$ (37.5 μL, 2 mg/mL in PBS, 1 eq) for 3 h at 25°C. Alternatively, if 2 eq of tetrazine-vcMMAE were used (1.5 μL, 1.4 mM in DMSO), they were mixed with 35 μL of PBS, and trastuzumab(CypK)$_2$ (37.5 μL, 2 mg/mL in PBS, 1 eq) for 20 h at 25°C.

In the conjugation time course, samples were analyzed by HPLC-HIC at each time point without further treatment. For other analyses and applications samples were purified using size-exclusion spin columns (Zeba™ spin desalting columns, 7K MWCO, 0.5 mL).

ADCs were characterized by HPLC-HIC (Thermo Scientific™ MAbPac™, HIC-20, 4.6 x 100 mm, 5 μm) with a flow of 1 mL/min, isocratic, with 100% A (1.5 M $(NH_4)_2SO_4$, 50 mM sodium phosphate pH 7.0, 5 % isopropanol) for 1 min followed by a 0-100% gradient of B (50 mM sodium phosphate pH 7.0, 20 % isopropanol) in A for 15 min. All chromatograms were acquired at 280 nm.

All antibodies were subjected to LC-MS analysis after overnight deglycosylation using PNGase F (NEB) at 37°C. A modified NanoAcquity (Waters, UK) delivered a flow of approximately 50 μl/min. A C4 UPLC BEH 1.7 μm, 2.1 x 5 mm pre-column (Waters,UK), trapped the proteins prior to separation on a C4 BEH 1.7 μm, 1.0 x 100 mm UPLC column. Proteins were eluted with a 20-minute gradient of acetonitrile (2% to 80%). The analytical column outlet was directly interfaced via an electrospray ionisation source, with a hybrid quadrupole time-of-flight (Q-TOF) mass spectrometer (Xevo G2, Waters, UK). Data was acquired over an m/z range of 350–4000, in positive ion mode with a cone voltage of 150v. Scans were summed together manually and deconvoluted using MaxEnt1 (Masslynx, Waters, UK). Intact antibodies were split into their heavy and light chains by reduction of disulfides with DTT in 8 M urea. The

proteins were subjected to LC-MS as above but data was acquired over an m/z range of 350-2000 with a cone voltage of 40 v.

To verify the incorporation of CypK at the target site, trastuzumab$(CypK)_2$ was digested with trypsin and analysed by tandem mass spectrometry. The antibody was reduced and alkylated in solution and digested with typsin or chymotrypsin at 1:20 ratio. The resulting peptides were analysed by nano-scale capillary LC-MS/MS using an Ultimate U3000 HPLC (ThermoScientific Dionex, San Jose, USA) to deliver a flow of approximately 300 nL/min. A C18 Acclaim PepMap100 5 μm, 100 μm x 20 mm nanoViper (ThermoScientific Dionex, San Jose, USA), trapped the peptides prior to separation on a C18 Acclaim PepMap100 3 μm, 75 μm x 250 mm nanoViper (ThermoScientific Dionex, San Jose, USA). Peptides were eluted with a 30 minute gradient of acetonitrile (2% to 60%). The analytical column outlet was directly interfaced via a nano-flow electrospray ionisation source, with a hybrid quadrupole orbitrap mass spectrometer (Q-Exactive Plus Orbitrap, ThermoScientific, San Jose, USA). Data dependent analysis was carried out, using a resolution of 30,000 for the full MS spectrum, followed by ten MS/MS spectra. MS spectra were collected over a m/z range of 300–2000. MS/MS scans were collected using a threshold energy of 27 for higher energy collisional dissociation (HCD). LC-MS/MS data were then searched against an in-house protein database which included SwisProt (UniProt KB) using the Mascot search engine programme (Matrix Science, UK).[4] Database search parameters were set with a precursor tolerance of 10 ppm and a fragment ion mass tolerance of 0.15 Da. One missed enzyme cleavage was allowed and variable modifications for oxidized methionine, carbamidomethyl cysteine, pyroglutamic acid, phosphorylated serine, threonine and tyrosine, and cypK. MS/MS data were validated using the Scaffold programme (Proteome Software Inc., USA).[5] All data were additionally interrogated manually.

All SDS-PAGE gels were run using NuPage™ 4-12% polyacrylamide gels (BioRad) and MES buffer.

HER2 binding assay. 96-well ELISA plates (Nunc MaxiSorp) were coated with HER2 (0.125 μg/mL) overnight at 4 ºC and blocked with 1% bovine serum albumin (BSA) for 1 h at room temperature (RT). Five washes with PBS with 0.05% tween (PBS-T) were performed after each step. For each sample, seven serial 2-fold dilutions were prepared from 100 ng/mL stock solutions. An anti-human antibody (W4038 Promega) conjugated to HRP was incubated (1:2500 in PBS-T with 0.5% BSA) 1 h at RT for detection. TMB (BioLegend) was used as HRP substrate and was allowed to react for 5-20 min before quenching with 1 M H_2SO_4. Background absorbance at 570 nm was subtracted from absorbance at 450 nm and a 4-parameter logistic regression regression was adjusted for each sample using Prism v.6 (GraphPad). All samples were run in triplicate.

Stability of the linkage in plasma and serum. The essay was performed as described by Junutula *et al.*[6] with some modifications. Briefly, 1 mL of serum or plasma was centrifuged for 5 min at 150 rcf at 4ºC and filtered through a 0.22 μm filter. 135 μL of serum or plasma was mixed with 15 μL of each trastuzumab(MMAE)$_2$ or trastuzumab(TAMRA)$_2$ (1 mg/mL in PBS) at a final concentration of 0.1 mg/mL. Samples were mixed thoroughly every day by pipetting up and down before aliquot extraction. 5 μL of each sample was extracted in triplicate every 24 h for 5 days, flash-frozen immediately, and stored at -80ºC.

All aliquots were thawed for quantification, diluted with PBS and analyzed using an indirect ELISA previously reported[7] with some modifications. Briefly, 96-well ELISA plates (Nunc MaxiSorp) were coated with HER2 (0.5 μg/mL) overnight at 4 ºC and blocked with 1 % bovine serum albumin (BSA) for 1 h at room temperature (RT). Five washes with PBS with 0.05% tween (PBS-T) were performed after every step. Samples were diluted 1:10000 in PBS and incubated for 2 h at RT. An anti-TAMRA antibody raised in mouse (ab171120 Abcam) was incubated (1:2000 in PBS-T with 0.5% BSA) for 1 h at RT. Finally, an anti-mouse HRP conjugate (sc-2005, Santa Cruz Biotechnology) was used for detection (1:1000). Controls with plasma and serum at day 0 and 5 were included in each plate. TMB (BioLegend) was used as HRP substrate and was allowed to react for 10-30 min before quenching with 1 M H_2SO_4. Background absorbance at 570 nm was subtracted from absorbance at 450 nm, a 5-parameter logistic

regression curve was adjusted to standard dilutions and the other samples (triplicates) were interpolated in this curve.

Fluorescence quantification was carried out by running samples diluted 1:100 with PBS on SDS-PAGE gels. Fluorescence intensity was measured by gel densitometry using Fiji.

Cytotoxicity assay. This assay was performed as previously reported.[8] Briefly, SK-BR-3 and MCF-7 cells were cultured in DMEM supplemented with 10% heat inactivated fetal bovine serum, 100 units/mL penicillin, and 100 ug/mL streptomycin. Cells were seeded in 96-well plates two days prior to treatment at 3000 cells in 100 μL/well. Two days later, each sample was diluted with 0.1% DMSO in DMEM, added in 100 μL aliquots and incubated for 5 days at 37ºC with 4% CO_2. At day 5, 125 μL of the medium containing the sample was removed and 75 μL of CellTiterGlo 2.0 (Promega) was added. After 10 min on an orbital shaker, 100 μL of the mixture was transferred to a 96-well white plate and the luminescence was read. The signal of each well was expressed as a percentage of control cells treated with 0.1% DMSO.

Supplementary figures and tables

Transient expression system

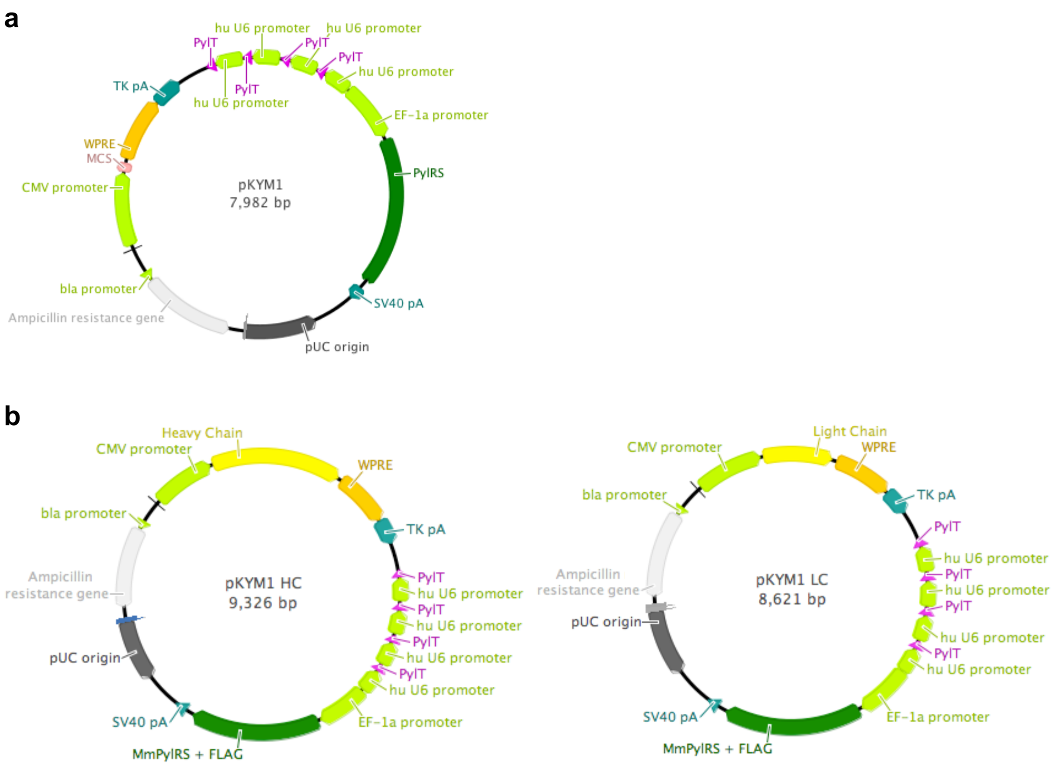

Figure S1. Plasmids for the transient expression system. pKym1 plasmid encoding *PylS*/*PylT* (a), which is based on pcDNA3.4, was used to generate the two plasmids (b) for transient expression. We utilized the strong constitutive CMV promoter and a WPRE sequence in the 3' untranslated region (UTR) known to increase mRNA stability and protein yield. The system comprises the constitutive EF1 α promoter driving PylS expression and four tandem cassettes with a U6 promoter driving Pol III transcription of PylT(U25C).

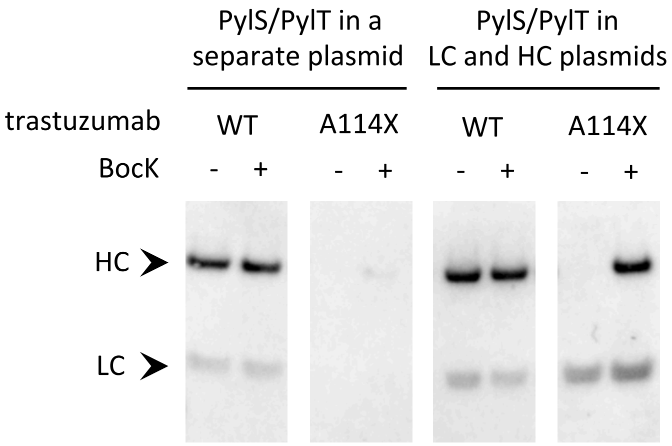

Figure S2. Western blot of trastuzumab expressed using two and three plasmids (anti-Human HRP W4038, Promega). Inserting the *PylS*/*PylT* cassette in the LC and HC plasmids allows substantially higher antibody expression levels than transfecting the LC, HC and PylS/PylT in three separate plasmids. Trastuzumab(A118X) refers to antibody with an amber stop codon at position HC-118, which is expected to be suppressed in the presence of ncAA (BocK in this case).

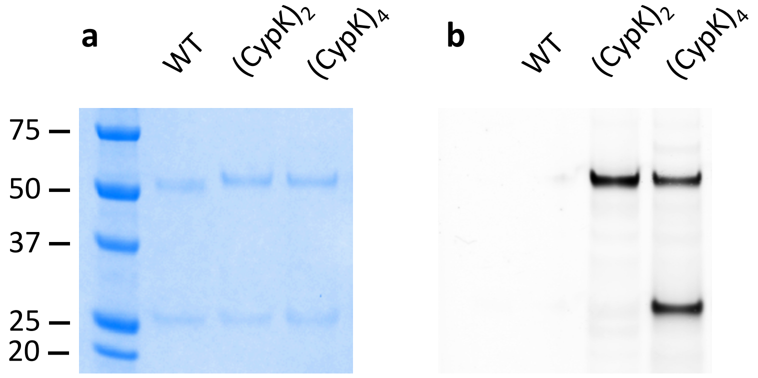

Figure S3. Trastuzumab can be easily expressed with 2 and 4 CypK handles per antibody molecule. These gels show the conjugation of tetrazine-TAMRA to trastuzumab. 0.3-0.5 μM trastuzumab(CypK)$_2$ or trastuzumab(CypK)$_4$ in PBS were incubated with 10 μM tetrazine-TAMRA at room temperature overnight. Trastuzuamb Wild Type (WT) was used as a reference in the SDS-PAGE gels. a) In the Direct-Blue-stained gel, the heavy and light chains in the three antibodies are present. A shift in the bands corresponding to the heavy chain of the CypK-containing antibodies indicates they have been modified. b) TAMRA in-gel fluorescence shows labeling of the heavy chains in trastuzumab(CypK)$_2$ and labeling of both light and heavy chains in trastuzumab(CypK)$_4$.

Stable expression cell lines

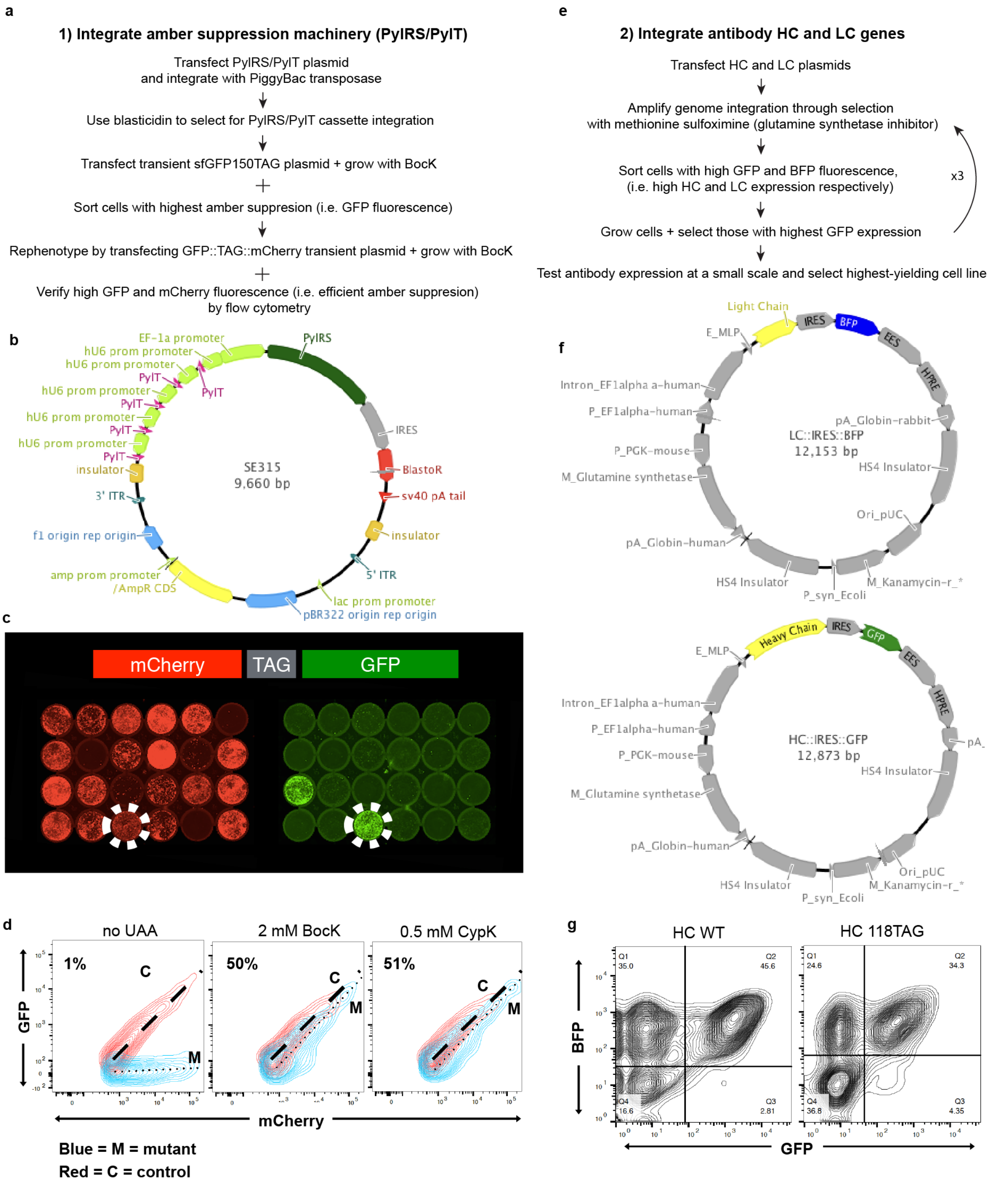

Figure S4. Generation of stable cell lines to produce antibodies bearing CypK. a) Diagram of the genomic integration of the amber suppression machinery. The amber suppressing line with *PylS* and *PylT* used in this study and generated following this procedure has shown stable expression for > 12 months of passaging. b) Plasmid containing the orthogonal translation machinery. c) Plates show mCherry and GFP fluorescence in the process of rephenotyping the amber suppressing lines using an GFP::TAG::mCherry plasmid. d) Flow cytometry density plots show GFP and mCherry expression in the presence and abscence of BocK and CypK. Using FlowJo, mCherry

positive cells were gated. We then took the arithmetic mean of GFP and divided by the arithmetic mean of mCherry to get a ratio. The ratio of mCherry-TAG-GFP over mCherry-GFP was taken as amber suppression efficiency. Remarkably, CypK was incorporated with the same efficiency as BocK as reflected by high GFP and mCherry correlation, which corresponds to 50% of a control without a non-stop between the two reporter proteins. Furthermore, only 1% of read-through is detected in the absence of ncAA. e) Diagram of the genomic integration of antibody heavy and light chains. f) Plasmids containing the light and heavy chains of the antibody. g) Flow cytometry density plots obtained when sorting the lines with highest expression of light and heavy chain, which are co-translationally epxressed with BFP and GFP, respectively.

a

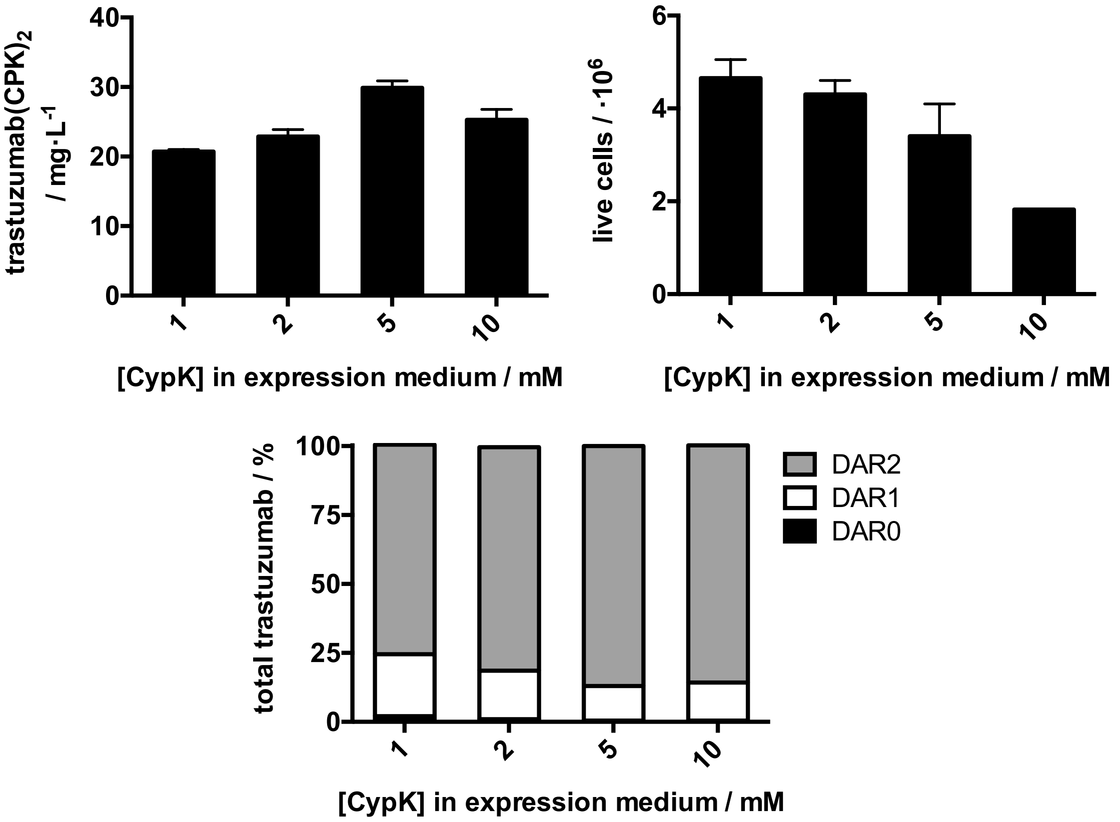

trastuzumab(CPK)$_2$ / mg·L^{-1}
40
30
20
10
0
1
2
5
10
[CypK] in expression medium / mM
live cells / ·10^6
6
4
2
0
1
2
5
10
[CypK] in expression medium / mM
total trastuzumab / %
100
75
50
25
0
1
2
5
10
[CypK] in expression medium / mM
DAR2
DAR1
DAR0

b

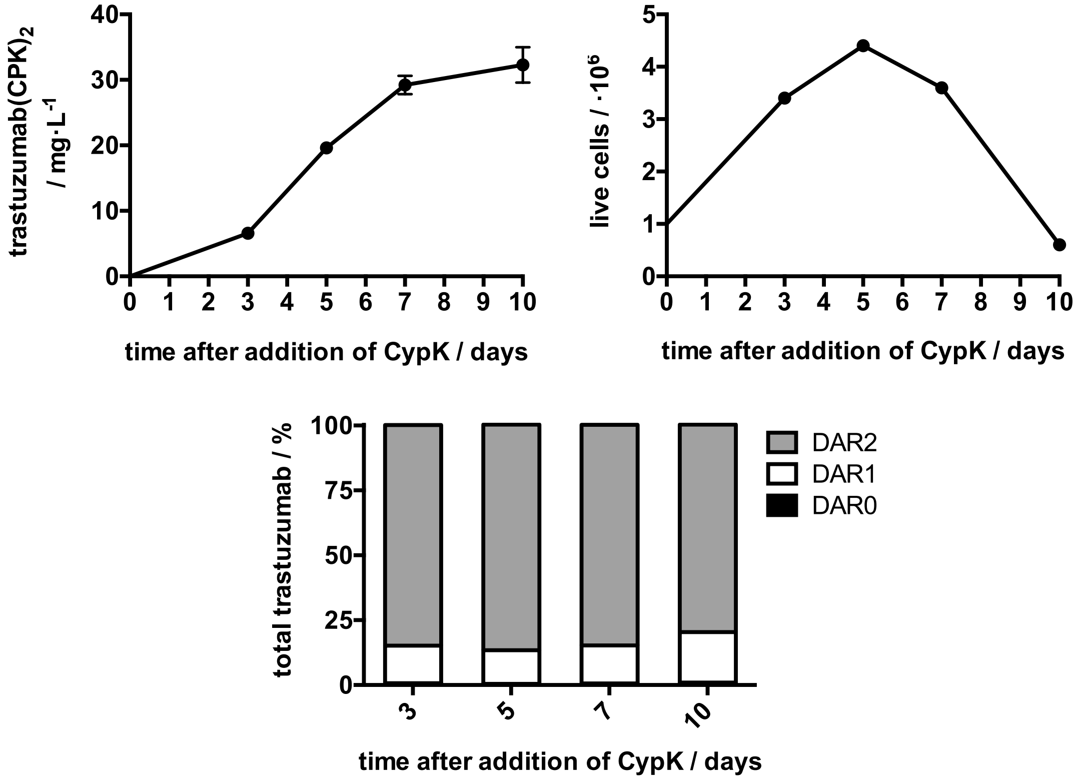

trastuzumab(CPK)$_2$ / mg·L^{-1}
40
30
20
10
0
0 1 2 3 4 5 6 7 8 9 10
time after addition of CypK / days
live cells / ·10^6
5
4
3
2
1
0
0 1 2 3 4 5 6 7 8 9 10
time after addition of CypK / days
total trastuzumab / %
100
75
50
25
0
3
5
7
10
time after addition of CypK / days
DAR2
DAR1
DAR0

Figure S5. Expression with 5 mM CypK for 5 days provides the highest DAR with tetrazine-cvMMAE. a) CHO-S HC-118TAG cell viability, antibody yield and percentage of species with each DAR at day 5 as a function of CypK concentration in expression medium. Cell viability is not affected at 10 mM CypK (data not shown), but cell growth decreases, thereby reducing the overall production of the antibody. CypK concentrations of 5-10 mM provide the highest levels of reactive handle in the antibody. b) CHO-S HC-118TAG cell viability, antibody yield after protein A purification and percentage of species with DAR 0, 1 and 2 as a function of time after CypK (5 mM) addition in expression medium. The content of reactive CypK in the protein is independent of the expression time until day 7 (Figure S7b), indicating that the amino acid is stable throughout this period. Cell viability was quantified by trypan blue staining, antibody yield was quantified after protein A purification using Direct Blue staining and relative abundance of DAR0, DAR1 and DAR2 was measured by HIC-HPLC-HIC. All error bars represent standard deviations of technical replicates.

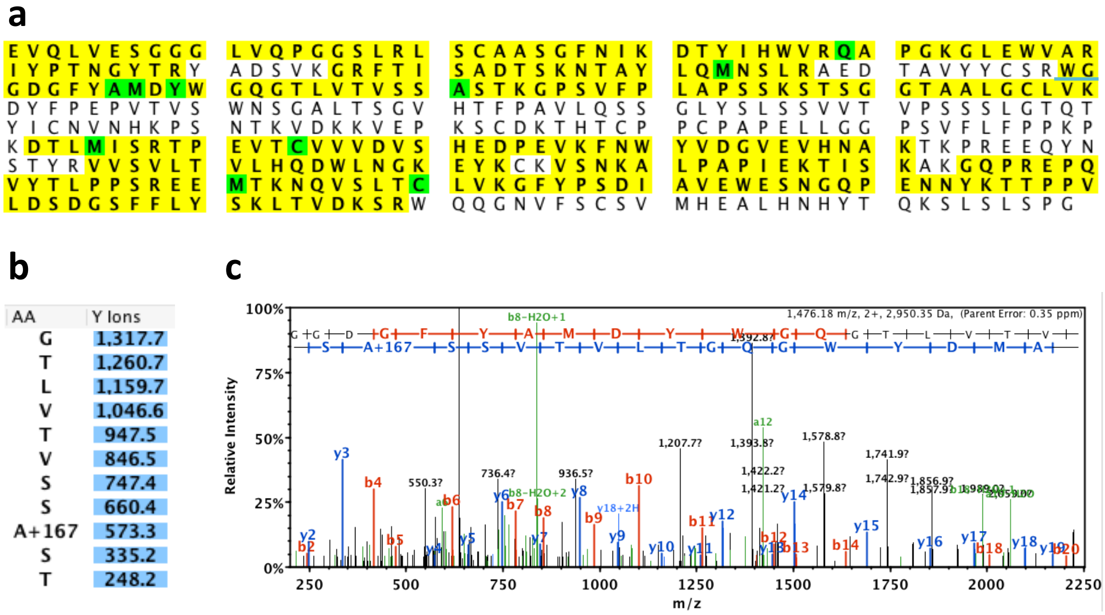

Figure S6. Tryptic digestion followed by tandem mass spectrometry confirms that CypK is incorporated in trastuzumab(CypK)$_2$ (5 mM CypK in expression medium). a) Peptide coverage map of trypsin-digested heavy chain. b) The *y* ions identified reveal that an amino acid with a mass 167 units heavier than alanine, i.e. CypK, has been incorporated in the target position. c) Fragmentation spectrum of the peptide including CypK (A+167).

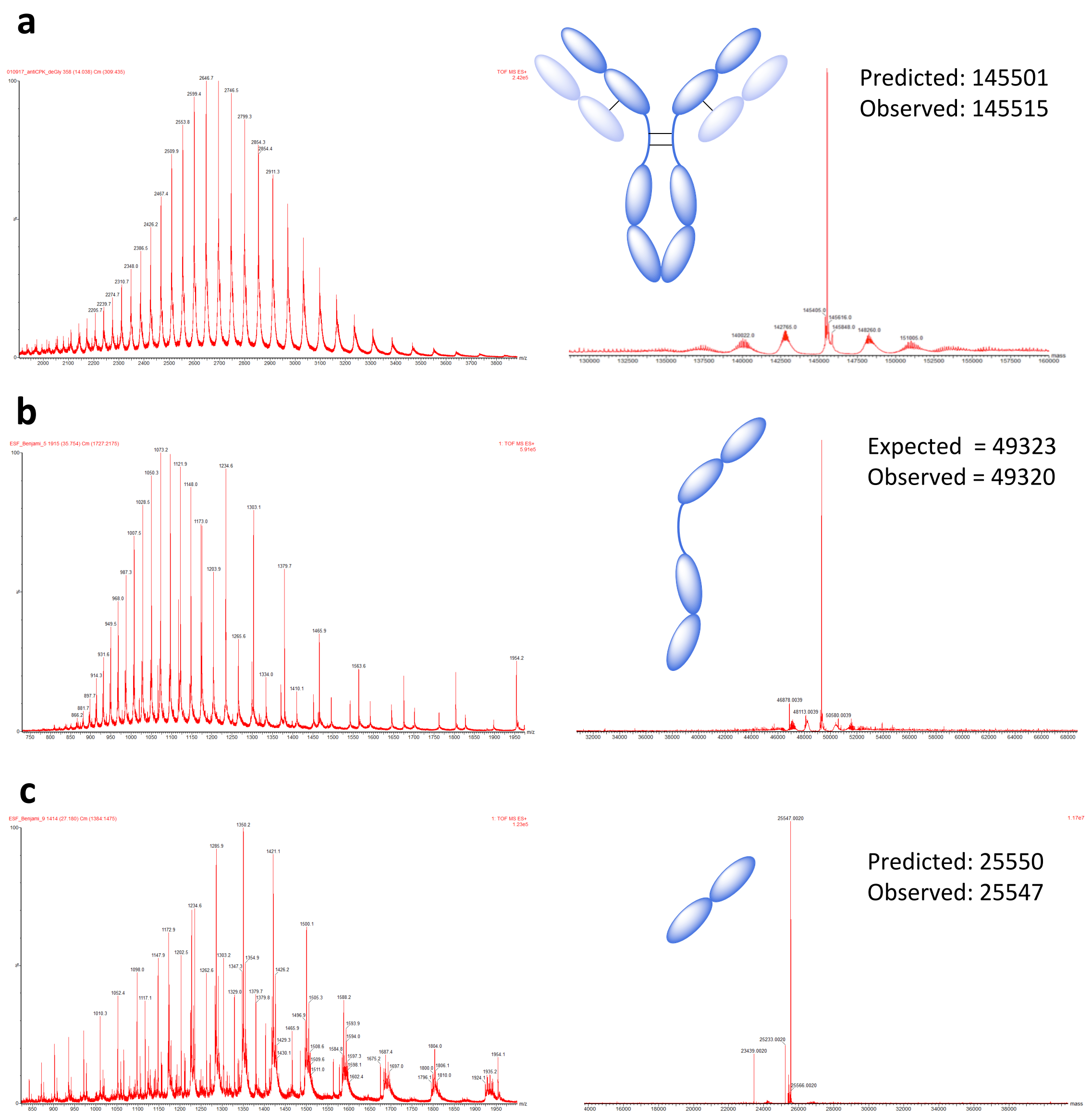

Figure S7. Mass spectrometry data proves the incorporation of CypK (5 mM CypK used for expression). Raw mass spectra of deglycosylated molecules are shown on the left and deconvoluted spectra on the right. a) Full antibody. b) Antibody reduced with DTT. c) VH-CH1 domains after digestion with IdES Fabricator™ (Genovis).

Antibody conjugation and analysis

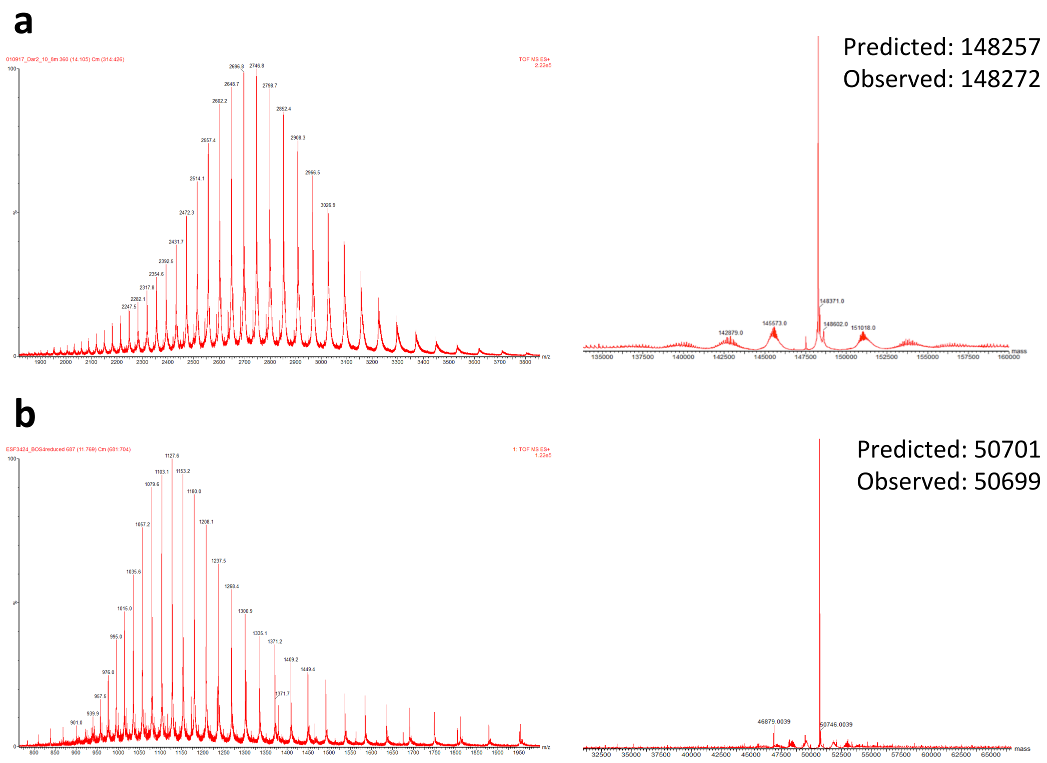

Figure S8. Mass spectrometry data proves the conjugation of tetrazine-vcMMAE to trastuzumab(CypK)$_2$. Raw mass spectra of deglycosylated molecules are shown on the left and deconvoluted spectra on the right. a) Full antibody. b) Antibody reduced with DTT.

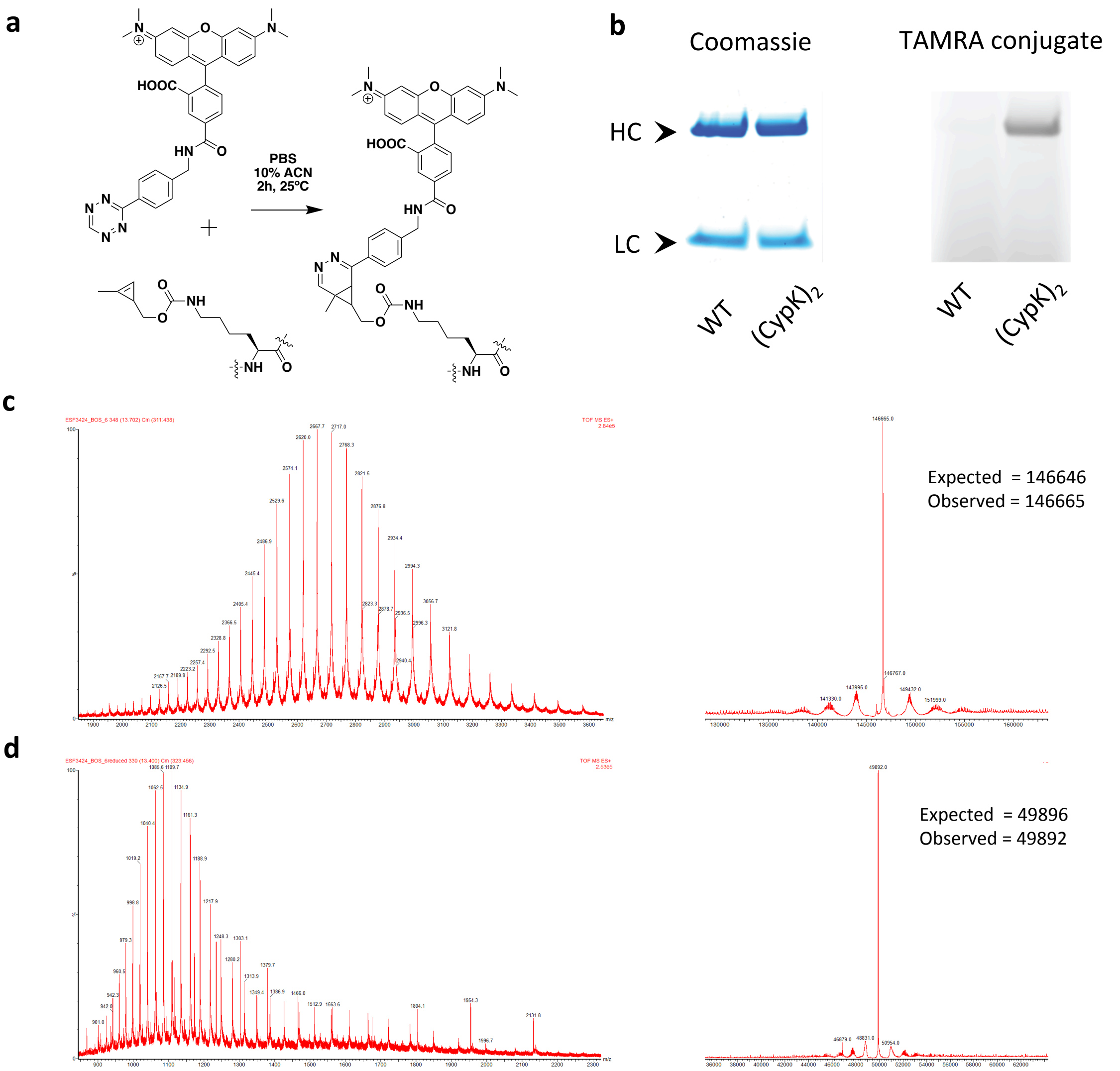

Figure S9. Conjugation of tetrazine-TAMRA to trastuzumab(CypK)$_2$. a) Reaction scheme. b) Direct-Blue-stained SDS-PAGE gel (left) and in-gel TAMRA fluorescence (right). c) Raw and deconvoluted mass spectra of full-length deglycosylated trastuzumab(TAMRA)$_2$ d) Raw and deconvoluted mass spectra of deglycosylated trastuzumab(TAMRA)$_2$ heavy chain after reduction with DTT.

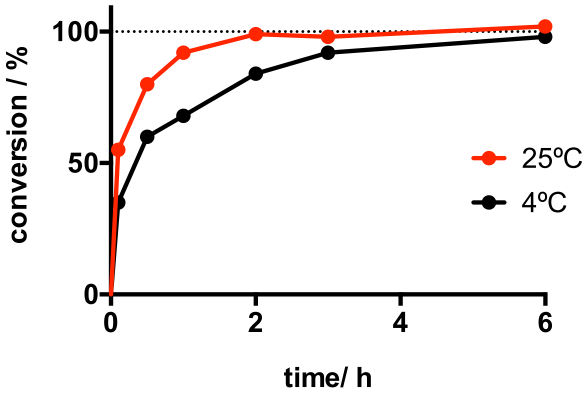

Figure S10. Reaction of trastuzumab(CypK)$_2$ with tetrazine-TAMRA takes less than 2h at room temperature. At 4ºC quantitative conjugation of tetrazine-TAMRA is reached within 3-6 h. Relative degree of conjugation was measured by in-gel fluorescence.

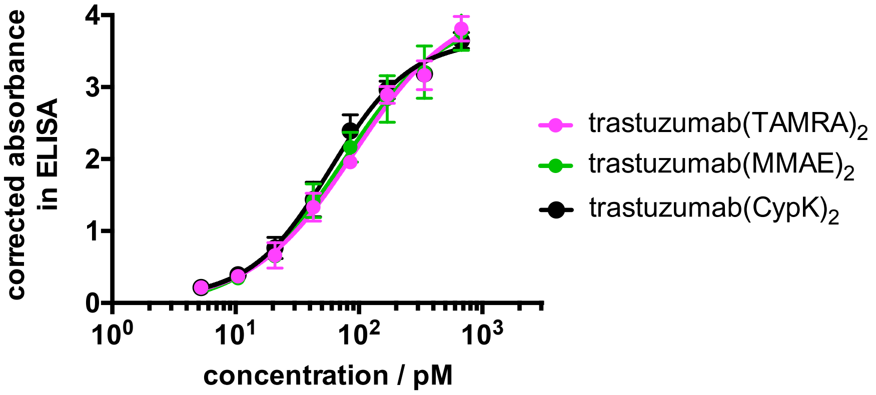

Figure S11. Trastuzumab(CypK)$_2$ preserves HER2 binding after conjugation with MMAE or TAMRA. HER2 (10004-HCCH Sino Biological) in PBS at 2.5 μg/mL was adsorbed on an ELISA plate. Samples were incubated after blocking with 1% BSA in PBS. Five washes with 0.1% tween 20 (PBST) were performed after each step. An anti-human-HRP conjugate (1:1000, anti-Human HRP W4038, Promega) was used for detection. Error bars represent the standard deviation of technical replicates.

Stability of the linkage in plasma and serum

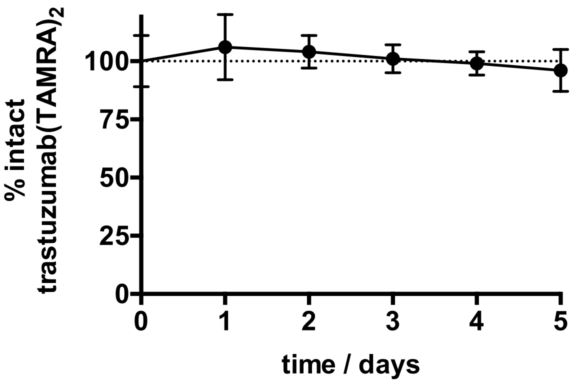

Figure S12. Trastuzumab(TAMRA)$_2$ is stable in human plasma at 37°C for greater than 5 days. Signal is measured by in-gel fluorescence of the antibody heavy chain. Error bars represent the standard deviation of biological replicates.

Cytotoxicity of trastuzumab-MMAE

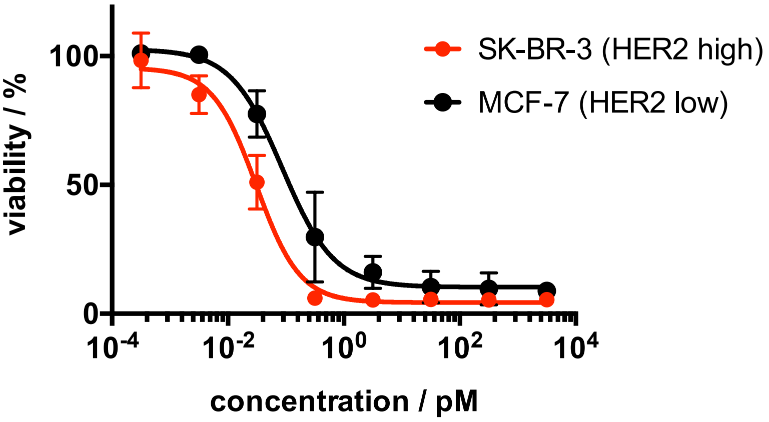

Figure S13. The toxicity of MMAE was found to be similar in the HER2+ and HER2- cell lines tested, which shows that the increased toxicity of trastuzumab(MMAE)$_2$ in SK-BR-3s is not due to a different effect of the toxin on the cells. The toxicity of the MMAE peptidomimetic toxin has previously reported to be cell-independent due to its membrane permeability, underscoring the requirement of attaching it to a targeting antibody to provide selectivity.[9] Furthermore, we observed that the toxicity of MMAE was 3-4 orders of magnitude higher than the toxin with the linker (tetrazine-vcMMAE, figures 3c and 3d in the main text).

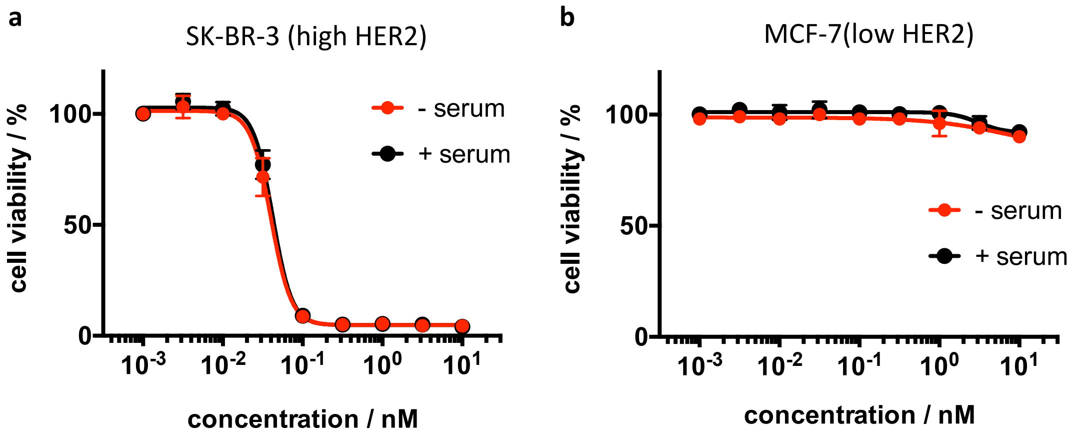

Figure S14. Viability of cells with high- (a) and low- (b) HER2 levels treated with trastuzuamb(MMAE)$_2$ is identical before and after incubation with human serum. The effect on SK-BR-3 (HER2 high) shows the ADC retains its potency, while the low toxicity in MCF-7 (HER2 low) implies absence of free drug. These results indicate that the linkage between trastuzumab and MMAE is stable under the essayed conditions. The ADC was first incubated for 5 days in 90% human serum and then added to cells (+ serum dataset). Trastuzuamb(MMAE)$_2$ freshly diluted in PBS (- serum dataset) was added in the same experiment for comparison. As described in the experimental section for the cytotoxicity assay, these compounds were incubated 5 days with cells and viability was assessed using CellTiter Glo 2.0. Higher concentrations of the ADC could not be used in these experiments in order to maintain the normal serum levels during the treatment for the viability essay. This is why the inflection point is not observed for MCF-7 in this experiment as opposed to the plot in Figure 3d in the main text. The error bars represent the standard deviation of biological triplicates.

	trastuzumab $(MMAE)_2$	trastuzumb $(CypK)_2$	trastuzumb WT	MMAE	tetrazine-vcMMAE
SK-BR3	0.055±0.010	-	-	0.031±0.008	24±5
MCF7	12±2	-	-	0.087±0.029	150±32

Table S1. EC_{50} (nM) for trastuzumab, the MMAE conjugate and the free peptides in the cytotoxicity assay. A 4-parameter logistic regression fitting was used to calculate EC_{50} on Prism v. 6 (GraphPad).

Trastuzumab sequence

A118 was mutated in trastuzumab(CypK)$_2$

A118 and K107 were mutated in trastuzumab(CypK)$_4$

Mutated residues are underlined

HC

EVQLVESGGGLVQPGGSLRLSCAASGFNIKDTYIHWVRQAPGKGLEWVARIYP
TNGYTRYADSVKGRFTISADTSKNTAYLQMNSLRAEDTAVYYCSRWGGDGFY
AMDYWGQGTLVTVSSASTKGPSVFPLAPSSKSTSGGTAALGCLVKDYFPEPVT
VSWNSGALTSGVHTFPAVLQSSGLYSLSSVVTVPSSSLGTQTYICNVNHKPSNT
KVDKKVEPKSCDKTHTCPPCPAPELLGGPSVFLFPPKPKDTLMISRTPEVTCVVV
DVSHEDPEVKFNWYVDGVEVHNAKTKPREEQYNSTYRVVSVLTVLHQDWLN
GKEYKCKVSNKALPAPIEKTISKAKGQPREPQVYTLPPSREEMTKNQVSLTCLV
KGFYPSDIAVEWESNGQPENNYKTTPPVLDSDGSFFLYSKLTVDKSRWQQGNV
FSCSVMHEALHNHYTQKSLSLSPG

LC

DIQMTQSPSSLSASVGDRVTITCRASQDVNTAVAWYQQKPGKAPKLLIYSASFL
YSGVPSRFSGSRSGTDFTLTISSLQPEDFATYYCQQHYTTPPTFGQGTKVEIKRT
VAAPSVFIFPPSDEQLKSGTASVVCLLNNFYPREAKVQWKVDNALQSGNSQES
VTEQDSKDSTYSLSSTLTLSKADYEKHKVYACEVTHQGLSSPVTKSFNRGEC

Supplementary References

1. W. H. Schmied, S. J. Elsässer, C. Uttamapinant and J. W. Chin, *J. Am. Chem. Soc.*, 2014, **136**, 15577-15583.
2. C. M. Hommelsheim, L. Frantzeskakis, M. Huang and B. Ülker, 2014, **4**, 5052.
3. T. S. Elliott, F. M. Townsley, A. Bianco, R. J. Ernst, A. Sachdeva, S. J. Elsässer, L. Davis, K. Lang, R. Pisa, S. Greiss, K. S. Lilley and J. W. Chin, *Nat. Biotech.*, 2014, **32**, 465-472.
4. D. N. Perkins, D. J. Pappin, D. M. Creasy and J. S. Cottrell, *Electrophoresis*, 1999, **20**, 3551-3567.
5. A. Keller, A. I. Nesvizhskii, E. Kolker and R. Aebersold, *Analytical Chemistry*, 2002, **74**, 5383-5392.
6. J. R. Junutula, H. Raab, S. Clark, S. Bhakta, D. D. Leipold, S. Weir, Y. Chen, M. Simpson, S. P. Tsai, M. S. Dennis*, et al.*, *Nat. Biotech.*, 2008, **26**, 925-932.
7. M. P. VanBrunt, K. Shanebeck, Z. Caldwell, J. Johnson, P. Thompson, T. Martin, H. Dong, G. Li, H. Xu, F. D'Hooge*, et al.*, *Bioconjug. Chem.*, 2015, **26**, 2249-2260.
8. Y. Shiraishi, T. Muramoto, K. Nagatomo, D. Shinmi, E. Honma, K. Masuda and M. Yamasaki, *Bioconjug. Chem.*, 2015, **26**, 1032-1040.
9. G. Badescu, P. Bryant, M. Bird, K. Henseleit, J. Swierkosz, V. Parekh, R. Tommasi, E. Pawlisz, K. Jurlewicz, M. Farys, N. Camper, X. Sheng, M. Fisher, R. Grygorash, A. Kyle, A. Abhilash, M. Frigerio, J. Edwards and A. Godwin, *Bioconjug. Chem.*, 2014, **25**, 1124-1136.
